# Right Unilateral Spatial Neglect Improves with Intrinsic Motivation

**DOI:** 10.1155/2022/4828549

**Published:** 2022-10-27

**Authors:** Hirotaka Saito, Haruka Kobayashi, Junichi Yatsu, Shigeru Obayashi

**Affiliations:** ^1^Department of Rehabilitation Medicine, Dokkyo Medical University Saitama Medical Center, Koshigaya, Japan; ^2^Department of Rehabilitation Medicine, Saitama Medical University International Medical Center, Hidaka, Japan

## Abstract

*Background*. In the acute phase of stroke, it is well known that the incidence and severity of unilateral spatial neglect (USN) are more significant in the right hemisphere injuries. Still, the detection of USN in left hemisphere injuries has been increasing in recent years. This trend is because behavioral assessments have prevented the exclusion of patients who are difficult to assess for USN or apathy using conventional paper-and-pencil tests (e.g., aphasia). Right USN and post-stroke apathy share many common lesions. Therefore, clinical symptoms may overlap, but little validation considers this. *Case Study*. A man (62 years old) determined to have the right USN and apathy was treated for six weeks in 3 terms. In the first term (weeks 1 to 2), the patient was treated for the right USN by conventional therapy. In the second term (3–4 weeks), treatment for right USN and apathy by goal-directed therapy based on affinity behavior was implemented. In the third term (5–6 weeks), goal-directed therapy based on affinity behavior was discontinued, and treatment was returned to conventional therapy only. In the second term (goal-directed therapy based on affinity behavior), the improvement in patients' apathy (clinical assessment for spontaneity) was more significant than the effect size in the third term (conventional therapy). There were no significant differences in USN (catherine bergego scale) and intrinsic motivation (pittsburgh rehabilitation participation scale). However, the effect size in the second term tended to be larger than in the third term (conventional therapy). *Clinical Rehabilitation Impact*. This report aims to demonstrate the limitations of current treatment for cases determined to have both right USN and apathy. Second, to assess the extent to which this new intervention can complement the limitations of current treatment.

## 1. Introduction

Unilateral spatial neglect (USN) is a common neurological syndrome in right hemisphere injuries, and the incidence of USN in acute stroke is more frequent in patients with left USN (right hemisphere injury) [1–3]. From studies half a century ago to the present, the detection of left USN has been consistently reported to be higher than that of right USN [4, 5], while the detection of right USN has been increasing in recent years [6–8]. This is presumably because it is generally challenging to assess USN with the conventional paper-and-pencil test in cases of left hemisphere injury with severe aphasia and paralysis of the dominant hand. The incidence of aphasia has been reported to be 68.9% in patients with right USN [9]. Therefore, USN may be overlooked in left hemisphere injuries due to aphasia. A behavioral assessment study reported that left and right USN incidence was above 55% and 37% [7, 8, 10], respectively, suggesting that the incidence of right USN may be higher than previously reported [9]. In addition, USN reportedly has a negative impact on functional recovery in rehabilitation outcomes regardless of whether the patient is on the left or right [1, 2]. Therefore, it is clinically important to evaluate and treat right USN in patients with left hemisphere injury, but there are few reports on the treatment of right USN [11–13].

According to a voxel-based lesion-symptom mapping report, in the right USN, lesions were observed in the left superior middle temporal gyrus, temporal pole, prefrontal cortex, and insular cortex; these were associated with severe USN [14]. This pattern of lesions in the right USN involves areas of the ventral attention system [15, 16] and partially reflects important areas of the right hemisphere known to be associated with the left USN. The ventral attention system controls stimulus-driven attention and responds to the unexpected occurrences of behaviorally relevant stimuli [15, 16]. It has been reported that patients with left hemisphere damage have no USN on neuropsychological tests (desk tests); however, right USN is evident in a spatial monitoring task with a demand for attentional load (multitasking) [17]. This suggests that the right USN comprises impaired stimulus-driven attention associated with the ventral attention system and an inability to control multiple environmental stimuli relevant to daily behavior. It has been proposed that USN is mainly related to dysfunction of attention and that potential factors affecting it may be related to motivation [18]. In recent years, it has become clear that attentional dysfunction in USN is modulated by reward-based motivational factors [19, 20]. Interestingly, there was no effect of exogenous dopamine (L-dopa) on USN across patients, and there was no evidence of a synergistic effect between L-dopa administration and reward-based motivation (endogenous dopamine) [19]. Also, reward-based motivation improved USN across patients, while those who did not respond to reward-based motivation showed USN-improvements in the reward task only after a single dose of L-dopa. Reward-based motivation has been shown to increase endogenous dopamine activity [21], and administration of exogenous dopamine may cause excessive dopamine levels in reward-responders. This suggests that optimal levels of dopamine in the brain are required to drive USN regulation and that suboptimal or supra-optimal levels are likely to impair it, in accordance with the report by Cools and D'Esposito [22]. Therefore, eliciting an increase in endogenous dopamine activity is important for the treatment of USN; however, so far, treatment patterns using reward-based motivation are scarce (e.g., monetary reward tasks).

Recently, it has been pointed out that post-stroke apathy is associated with reward sensitivity [23]. Apathy is defined primarily as a motivational disorder, believed to be a decline in goal-directed behavior and cognition, but consensus on the definition of apathy has not yet been achieved [24]. In a report by Rochat et al. [23] investigating the relationship between reward sensitivity and apathy after stroke, it was observed that apathy scores increased with decreasing reward sensitivity and confirmed the association of the bilateral dorsal striatum, dorsal thalamus, left prefrontal cortex, and insular cortex with increased apathy. Many of these apathy-related lesions are consistent with those associated with the right USN of Beume et al. [14] and may be associated with the region responsible for the control of stimulus-driven attention by the ventral attention system [15, 16].

There is a growing consensus on the effectiveness of a combination of bottom-up and top-down approaches for USN [25]. However, the top-down approach is difficult for patients with impaired awareness of their USN symptoms and spatial deficits, that is, USN anosognosia [26], or for those with difficulties in sustaining attention to a specific task for a certain period of time [27]. These patients are presumed to have difficulties actively participating in treatment, and the same may be true for the right USN patients with poststroke apathy. Reportedly, treatment of USN patients with impaired disability awareness using meaningful goal-directed tasks may improve disability awareness, USN and the ability to perform daily activities [28, 29]. Therefore, in the right USN patients with post-stroke apathy, conventional top-down approaches may fail due to decreased reward sensitivity; for such cases, treatments that induce the patients' goals and reward prediction are required. However, to our knowledge, there are few reports describing the treatment of right USN with apathy.

The decreased reward sensitivity associated with poststroke apathy and the decreased stimulus-driven attention due to the impairment of the ventral attention system may influence each other and be expressed as the right USN. Because these factors of the right USN are associated with impaired motivation and treatment should focus on motivation. Demonstrating the effects of motivation-focused treatment may contribute to USN patients who cannot participate in treatment due to motivational problems. Therefore, this case study aims to report the effect of a motivational-focused treatment on the right USN with apathy compared to the current traditional treatment.

## 2. Case Report

### 2.1. Findings on Admission

**Figure 1:**
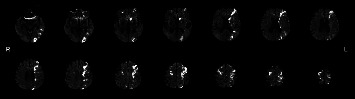
Brain images of the study patient. Diffusion-weighted magnetic resonance images of the patient (day 3 from stroke onset). Multiple infarctions in the bilateral cerebrum were observed, mainly in the left parietal and occipital cortex/bilateral prefrontal cortex, as cortical lesions basal ganglia (left caudate nucleus) were presented as a subcortical lesion.

The patient is a 62-year-old right-handed man whose ischemic stroke affected both cerebral hemispheres, with the left side being more involved (Figure 1). A few days after the stroke, the patient awakened with eyes open but showed right hemiparesis, decreased perception of the right space (e.g., prominent deviation of the head and gaze to the left space), and decreased spontaneous responses. Previously a hard-working, diplomatic person, the patient had become taciturn and reserved, and showed a lack of interest in external stimuli and others, along with reduced spontaneity in behavior and thought.

Clinically, the patient had difficulty moving the right side of his body and did not attempt to see the right visual space. He had impaired consciousness in verbal and motor responses (Glasgow Coma Scale, E4/V1/M4) and severe motor paralysis (Stroke Impairment Assessment Set-motor, 0/25) (Table 1).

In addition, the patient had difficulty speaking and did not respond to other people's questions or stimuli, and did not ask any questions. The patient was awake with open eyes and lacked response to external stimuli (e.g., body shaking), although he was occasionally observed moving the left side of his body (non-paralyzed side) by himself. Therefore, it was difficult to ask patients to what extent these conditions were affected by aphasia or apathy. In other words, assessment of aphasia and apathy using the gold standard questionnaire was difficult. This was also true for the assessment of cognitive and USN function. Thus, we used the SIAS-Speech and clinical assessment for spontaneity (CAS) interview-based motivation scale to assess aphasia and apathy, respectively. The SIAS-Speech assesses deficits in expressive and comprehension skills [30]. A score of 0 indicated overall aphasia, 1 indicated moderate aphasia, 2 indicated mild aphasia, and 3 indicated no signs of aphasia (Table 1). The patient was aphasic (0/3; scores ≤2 were aphasic) and showed severe apathy (58/60; scores ≥3 were apathetic, Table 1). In addition, as apathy is defined primarily as a disorder of motivation [24], the patient was evaluated according to the Pittsburgh Rehabilitation Participation Scale (PRPS) to assess the degree of motivation. The patient had very low motivation (2.33/6 points, Table 1). The Catherine Bergego Scale (CBS) was used to assess USN because of the difficulty of using a conventional paper-and-pencil test [31]. The patient was considered to have severe USN (30/30; scores ≥21 indicate severe USN, Table 1). In subacute stroke, the CBS has a high sensitivity to the right USN [7,8] and correlates with conventional paper-and-pencil testing [32]. CBS, CAS, and PRPS were performed at every session, and the total number of measurements in all procedures was 30. This patient showed severe USN and an inability to control environmental stimuli (stimulus-driven attention) relevant to daily behavior. The patient did not respond to any visual, tactile, or auditory sensory stimulation from environmental stimuli. This reduction in stimulus-driven attention is due to a reduction in the ventral attentional system. However, visual search during feeding assistance and restlessness during voiding were frequently observed. Therefore, USN in this patient may also be related to a reduction in reward sensitivity due to apathy.

The purpose of this study was explained to the participant and his family in accordance with legal international regulations (Declaration of Helsinki, 1964). Case reports are exempt from review by the Ethics Committee of our hospital. Early mobilization was implemented on the second day after onset, and detailed evaluation and USN treatment were initiated on the seventh day after onset.

### 2.2. Outcome Measures

#### 2.2.1. Catherine Bergego Scale

The CBS is an assessment tool developed to evaluate USN symptoms in terms of activities of daily living [33]. It consists of 10 items of daily activity. It is more sensitive than the paper-and-pencil test and has higher reliability and validity [34]. CBS is assessed by observing the behavior of USN patients and, thus, includes patients with paralysis or aphasia of the dominant hand, who are generally excluded from the conventional paper-and-pencil USN test [7–9]. It has also been reported to be highly sensitive to the right USN [7–9]. The score for each item in the CBS ranges from 0 (no USN) to 3 (severe USN), for a total of 30. A score of 1–10 is considered mild USN, while 11–20 is considered moderate USN, and 21–30 is considered severe USN. The therapist who provided the intervention evaluated the patient using the CBS.

#### 2.2.2. Clinical Assessment for Spontaneity

The CAS is a standardized tool for the evaluation of apathy in Japan and consists of five tests (interview assessment, self-assessment, caregiver assessment, daily behavioral observation, and comprehensive clinical assessment) [35]. The CAS has been developed for Japanese people, and its reliability has been confirmed. The observation-based assessment of CAS can be adapted to patients with severe apathy and aphasia. In this study, the interview assessment conducted on the patient was evaluated by a therapist. Measurements were taken at every session. The interview assessment comprised 15 items, each with a score ranging from 0 (normal) to 5 (severely impaired), which were rated directly by the examiner. The total score range was 0–60, with a higher score indicating severe apathy. The cutoff value was 3 (in the 60 s), and a score ≥3 indicated apathy.

#### 2.2.3. Pittsburgh Rehabilitation Participation Scale

The PRPS is a 6-point Likert scale developed to assess patient participation during therapy sessions [36]. Studies using antigraphy as an objective measure have found that PRPS is a reliable indicator of participation during therapy sessions [37] with a high degree of validity [36]. The scores ranged from 0–6, with higher scores indicating that participants actively participated in the therapy. Assessments were recorded by each of the assigned therapists (i.e., occupational therapists, physical therapists, and speech therapists) at each session during the intervention period. The average score for the day was calculated by summing the scores obtained from each therapist and dividing them by the number of therapists involved.

### 2.3. Intervention and Management

**Figure 2:**
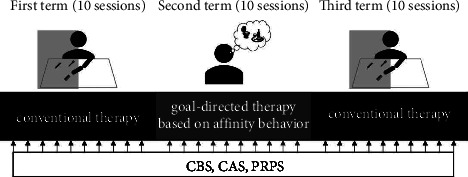
Experimental study procedure. CBS: catherine bergego scale, CAS: clinical assessment for spontaneity, and PRPS: pittsburgh rehabilitation participation scale.

In the report, we used a single-case experimental design (ABA design) to examine the effects of goal-directed therapy based on affinity behavior on the right USN with apathy. In the first term, the patient received conventional therapy. Goal-directed therapy based on affinity behavior was implemented in the second term. Finally, in the third term, goal-directed therapy based on affinity behaviors was discontinued, and the treatment was returned to conventional therapy alone. Each term lasted for two weeks (40 min/day, five days per week). The entire process took six weeks (Figure 2).

#### 2.3.1. Conventional Therapy

This was performed in the first and third terms. Conventional therapy was implemented by referring to the “figure description” of visual scanning training [38], which is the most used in the top-down approach [39]. During treatment, the patient attempted to learn visual searching by placing a visually salient stimulus (e.g., a brightly colored object) at the edge of a defined workspace (e.g., a table) and was instructed to look for an item on the table in front of him while sitting in a wheelchair. The patient's variety of items were used and placed in the right visual space to encourage visual exploration. Difficulties in visual exploration of the right visual space were guided by the therapist, who used specific strategies to guide the patient based on his performance on the spatial task. This guidance was in the form of supportive feedback and assistance to self-detect omission errors [40]. The purpose was to teach the patient exploratory strategies to help improve impairment recognition and promote a sense of control over the USN symptoms [41]. We also incorporated the neurorehabilitation principle of repetition (i.e., multiple practices in one session) and varied the task characteristics (i.e., size of the search space and distraction items).

#### 2.3.2. Goal-Directed Therapy Based on Affinity Behavior

This was performed during the second term. The therapist found meaningful behaviors (i.e., affinity behaviors) for the patient in everyday behavioral contexts and set them as goal-directed tasks based on affinity. The purpose was to elicit the patient's intrinsic motivation to help with goal-directed behavior and to regulate the USN. Prior to the second term, information on the patient's daily activities was collected to determine their affinity behavior. The patient had severe motor paralysis and spent most of the time in bed. However, there were opportunities to encounter external stimuli during daily life events (e.g., eating, changing clothes, defecating). The patient was noted to be severely apathetic, and visual search during feeding assistance and restlessness during voiding were frequently observed. We believed that these responses were spontaneous and an indicator of intrinsic motivation. We assessed the influence of the elicited intrinsic motivation on goal-directed behavior and adopted it as a goal-directed task based on affinity behavior with PRPS ≥3 (PRPS = 3 is moderate participation in the behavior). In other words, we judged affinity behaviors (i.e., meaningful to the patient) as behaviors in which the patient showed voluntary attention and goal direction in daily life. The behavior was then set as a context-dependent goal-directed task that elicited intrinsic motivation, and we attempted to determine whether goal-directed therapy incorporating the task would improve apathy and right USN.

The interventions involved goal-directed therapy in eating and elimination activities using intrinsic motivation. To effectively use patient's limited intrinsic motivation to aid goal-directed behavior, information was shared with the staff member responsible for patient's meal care (i.e., a speech therapist), and sessions of goal-directed therapy based on meal-based affinity behavior were timed to coincide with lunchtime. In addition, goal-directed therapy based on affinity behavior during defecation was also conducted by guiding the patient to a toilet space to promote goal-directed behavior and visual exploration. In the case of a patient with severe motor paralysis such as the patient, defecation would normally have to be performed in bed; however, to preserve the patient's intrinsic motivation, it was performed with physical assistance. While it was desirable to implement the intervention at the time of the desire to defecate, it was difficult to do so considering the unpredictability of the desire to defecate. Thus, the intervention was implemented at the time of the therapist's intervention, regardless of its presence or absence. After the goal-directed tasks based on affinity (i.e., eating and elimination) were conducted, additional goal-oriented tasks (i.e., wiping one's mouth, applying toothpaste, and washing one's hands) derived from the context of that task were performed. In the goal-directed therapies based on affinity behavior (i.e., eating and elimination) and goal-oriented tasks (i.e., wiping one's mouth, applying toothpaste, and washing one's hands), the patient was guided in visually exploring the right visual space by the therapist, similar to the guidance for conventional therapy [40, 41].

### 2.4. Statistical Analysis

Comparison of the measurements of the three terms was performed using the Bonferroni-corrected Wilcoxon test after finding statistical significance (*p* < 0.01) with the Friedman test. The statistical significance of the Bonferroni-corrected Wilcoxon test was set at *p* < 0.0167. The Tau-U was used to calculate the effect size in conventional therapy and goal-directed therapy based on affinity behavior [42]. Also, the relationship between the improvement in right USN, apathy, and intrinsic motivation was confirmed using Spearman's correlation coefficient. The statistical significance of the effect size and correlation coefficient was set at *p* < 0.05. The statistical analyses performed multiple comparisons and correlation analyses using R [43], and effect sizes were calculated using an online web-based calculator [44].

### 2.5. Results

The patient showed improvement in GCS verbal and motor responses during the study. The patient had a GCS verbal response score of 1 (no speech) until the second term but improved to a verbal response score of 4 (conversational) in the third term. Although the conversation was somewhat imprecise, the patient gradually increased his response to questions. Therefore, the third term was judged to be 6 (moving the limbs according to commands) on the GCS motor response score and 1 (moderate) on the SIAS-Speech score. In other words, he may have been assessed as having severe aphasia due to apathy. Motor paralysis did not improve much during the study, and severe paralysis persisted.

#### 2.5.1. CBS

**Figure 3:**
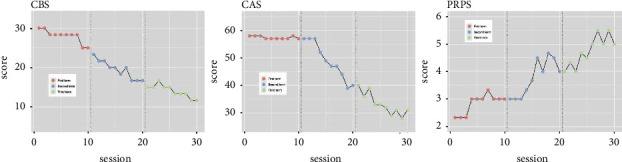
Trend of outcomes in each term. The downslope of CBS and CAS indicate an improving trend. The upslope of PRPS indicate an improving trend. CBS: catherine bergego scale, CAS: clinical assessment for spontaneity, and PRPS: pittsburgh rehabilitation participation scale.

The CBS scores for the first, second, and third terms are shown in Table 2. The score in the second term was significantly lower than that in the first term (*p*=0.015), and the score in the third term was significantly lower than that in the second term (*p*=0.014). In addition, the effect sizes of CBS scores in the first, second, and third terms are shown in Table 3. The effect size in the second term was significantly larger than that in the first term (Tau-U = −0.72, *p*=0.007, 90% CI [−1, −0.285], large change), and the difference in effect size between the second and first terms was larger than that between the third and second terms (Tau-U = −0.61, *p*=0.021, 90% CI [−1, −0.175], large change). Both term comparisons and effect sizes were comparable, but the trend of improvement was significant in the second term (Table 3, Figure 3).

#### 2.5.2. CAS

The CAS scores for the first, second, and third terms are shown in Table 2. There was no significant difference between the scores of the second and first terms (*p*=0.017) or the third and second terms (*p*=0.018). In addition, the effect sizes of CAS scores in the first, second, and third terms are shown in Table 3. The effect size in the second term was significantly larger than in the first term (Tau-U = −0.68, *p*=0.01, 90% CI [−1, −0.245], large change), and the difference in effect size between the second and first terms was larger than that between the third and second terms (Tau-U = −0.57, *p*=0.031, 90% CI [−1, −0.135], moderate change). Term comparisons were comparable, but effect sizes were more significant in the second term (Table 3, Figure 3).

#### 2.5.3. PRPS

The PRPS scores for the first, second, and third terms are shown in Table 2. There was a significant difference between the PRPS scores of the second and first terms (*p*=0.016), but not between the third and second terms (*p*=0.017). The effect sizes of PRPS scores in the first, second, and third terms are shown in Table 3. The effect size in the second term was significantly larger than that in the first term (Tau-U = 0.54, *p*=0.041, 90% CI [0.105–0.975], moderate change). The third term did not show a significant effect size (Tau-U = 0.43, *p*=0.104, 90% CI [−0.005–0.865]). Term comparisons and effect sizes were more significant in the second term (Table 3, Figure 3).

#### 2.5.4. Correlation

The correlations between the CBS and CAS scores and between the CAS and PRPS scores are shown in Table 4. There was a significant positive correlation between improvement in CBS score and improvement in CAS score (*r* = 0.96, *p* < 0.001, 95% CI [0.909, 0.979]). There was also a significant negative correlation between improvement in CBS score and improvement in PRPS score (*r* = −0.93, *p* < 0.001, 95% CI [–0.968, –0.863]). Furthermore, there was a significant negative correlation between improvement in CAS score and improvement in PRPS score (*r* = −0.96, *p* < 0.001, 95% CI [–0.981, –0.917]). Correlations were found for all outcomes, and USN seemed to be associated with motivational problems.

## 3. Discussion

This case study aimed to report the efficacy of a motivational-focused treatment compared to conventional treatment for right USN with apathy. This is because the right USN may be expressed by the reciprocal effects of reduced reward sensitivity associated with post-stroke apathy and the decreased stimulus-driven attention due to the impairment of the ventral attention system. Motivational-based treatment is also clinically meaningful because it may contribute to patients with USN who cannot participate in treatment due to motivational problems. The present results provide some novelties. First, improvement in right USN was associated with improvement in apathy. The effect size of CBS was similar in the second and third terms but was more significant in the second term when the effect size of CAS was greater. Second, improvements in intrinsic motivation seemed to be associated with improvements in right USN and apathy. In particular, the relationship between intrinsic motivation and apathy seemed to be strong. The effect size of PRPS in the second term, when the effect of intrinsic motivation was observed, was more significant than in the third term. These findings suggest that goal-directed therapy based on affinity behavior may be able to contribute to patients with the right USN.

In this report, in the treatment of USN, where reward-based motivation is important for voluntary attention [45, 46], we found improvements in both right USN and apathy, despite comorbid apathy causing reduced reward sensitivity [23] and goal-directed behavior [24]. Previous studies on the treatment of USN have reported that USN improved with a top-down approach (visual exploration training) and a bottom-up approach (cervical vibration stimulation and prism adaptation), whereas the top-down approach alone showed slight improvement [47, 48]. Goal-directed therapy based on affinity behavior in this report was also supported by explicit guidance by the therapist (top-down approach) and stimulation of objects and tasks (bottom-up approach) when visual exploration of the right visual space was difficult. In other words, it was a treatment that combined the top-down and bottom-up approaches, but it differed in that the voluntary attention required in the USN treatment was elicited through reward-based intrinsic motivation. Conventional therapy (top-down approach), which has a motivation-based goal-directed behavior component, involves learning through repeated visual search with explicit guidance, in which participants are instructed to look for visually salient stimuli that are intentionally placed in a defined workspace (e.g., a table) [39]. This is largely due to goal-directed behavior being based on extrinsic motivation.

Extrinsic motivation involves the expectation of material or social considerations in performing a task [49], while intrinsic motivation is generated from interests, concerns, or acts on the task itself [50]. In recent years, several reports have enumerated the importance of intrinsic motivation for learning [51, 52], and intrinsic motivation has been explained by neuroscience based on the desire to explore; dopaminergic systems are reportedly involved in intrinsic motivation [53]. This supports reports showing reward-based motivation leads to an increase in endogenous dopamine activity [21]. Intrinsic motivation mainly involves the ventral striatum [54], which helps maintain interest and persistence in the absence of external rewards by responding to positive or negative feedback elicited in the task-specific context [55]. As a neural basis, intrinsic motivation is supported by the tonic activity of dopamine, and based on this, we respond to environmental information with phasic activity [56]. This correlates with previous reports on dopamine in the brain acting in an inverted U-shape on cognitive control, thus requiring optimal levels in response to environmental information [22]. Marsden et al. [56] reported that the amygdala, parahippocampal gyrus, insula, anterior cingulate cortex, and caudate nucleus regions were involved with intrinsic motivation, which is consistent with reports investigating the relationship between intrinsic motivation and the dopaminergic system [57, 58]. These elements have been described as salience networks, which are neural systems that support intrinsic motivation [59] and may support the mobilization of attentional resources necessary for goal-directed behavior and reward prediction.

In this report, the patient's lesions were caused by multiple infarcts and were located in the bilateral prefrontal cortex and left caudate nucleus. The locations of these lesions were similar to those of the right USN [14] and poststroke apathy [23]. These lesions have a lot in common with the neural basis of intrinsic motivation [56–59]. Therefore, it is assumed that the patient had clinical symptoms of right USN and apathy because of the associated lesions. The detection of right USN is on the rise [6–8] and adversely affects functional recovery in rehabilitation outcomes regardless of left or right [1, 2]. There is a report that the right USN was milder and recovered more quickly than the left USN [60], but the evaluation of USN in this report relies on the patient's spontaneous response. Therefore, it should be noted that patients who do not respond are excluded. In other words, severe right USN may not be included. Damasio et al. reported severe right USN and akinetic mutism due to lesions mainly in the left prefrontal cortex, which improved with improvement in spontaneity [61]. They further mention that bilateral prefrontal lesions are prone to severe USN. The patient also showed severe right USN and apathy, such as akinetic mutism, but USN and apathy improved with improved intrinsic motivation (i.e., spontaneity). The location of the lesions was also similar. These findings suggest that the outcome may have been influenced by promoting patients' intrinsic motivation through goal-directed therapy based on affinity behavior.

Goal-directed training should be conducted in a natural environment whenever possible, as the performance of the task depends on the context created by the environment [62]. In addition, intrinsically motivating activities are those that provide manageable tasks, clear proximal goals, and immediate feedback [63]. Reward-based motivation is important in the treatment of USN requiring voluntary attention [45, 46], which is triggered by an individual's goals and reward predictions [64], which elicit adaptive responses in task performance [65, 66]. On the other hand, voluntary attention triggered by nonselfish goals can disrupt the control of spatial attention [64] and motivation based on external rewards has been shown to undermine intrinsic motivation [67].

Therefore, it is difficult to treat a patient who has decreased stimulus-driven attention due to right USN [15, 16] and decreased reward sensitivity due to apathy [23] with the conventional top-down approach, and treatment based on intrinsic motivation to induce voluntary attention by self-goals and reward prediction is necessary. Also, because reward sensitivity has much in common with the neural basis of intrinsic motivation [56–59], a goal-directed task in a high-affinity behavioral context performed on the present patient may provide important information about effective treatments for right USN.

## 4. Conclusion

Alternative and advanced interventions are needed to improve the neurological symptoms of stroke patients with motivational problems. This case study showed that motivational-based treatment could improve daily neglected symptoms and apathy in patients with right USN, although generalizability is limited.

## Figures and Tables

**Table 1:** Participant attributes.

Age and gender	Type of injury	Affected side	Time after onset (day)	GCS^#^	Severity of motor impairment^*∗*^^†^	Severity ofaphasia^*∗*^	Cognitive function	CBS	CAS	PRPS
62 years M	Embolic	Right	7	4/1/4	0,0/0,0,0 (severe)	0 (severe)	NM	30 (severe)	58 (severe)	2.33 (low motivation)

GCS: glasgow coma scale, CBS: catherine bergego scale, CAS: clinical assessment for spontaneity, PRPS: pittsburgh rehabilitation participation scale, NM: nonmeasurable. ^#^GCS; eye opening/verbal response/motor response.  ^*∗*^Evaluated by stroke impairment assessment set (SIAS). ^†^SIAS-motor; knee-mouth, finger-function/hip-flexion, knee-extension, foot-pat.

**Table 2:** Comparison of outcomes in each term.

	First term median (IQR)	First to second term		Second to third term	
		Second term median (IQR)	*p* value	Third term median (IQR)	*p* value
CBS	28.32 (28.32, 28.32)	20 (17.09, 21.26)	0.015^*∗*^	14.16 (13.32, 15)	0.014^*∗*^
CAS	57 (57, 58)	48 (44.75, 55.75)	0.017	32.5 (31, 35.25)	0.018
PRPS	3 (2.5, 3)	3.83 (3.08, 4.38)	0.016^*∗*^	4.83 (4.38, 5)	0.017

CBS: catherine bergego scale, CAS: clinical assessment for spontaneity, PRPS: pittsburgh rehabilitation participation scale. Bonferroni-corrected wilcoxon test for multiple comparisons was applied (adjusted ^*∗*^*p* < 0.0167).

**Table 3:** Tau-U effect sizes for each term change in the outcomes.

	First to second term					Second to third term				
	Tau-U	*p* value	90% CI		Effect size	Tau-U	*p* value	90% CI		Effect size
CBS	−0.72	0.007^†^	−1	−0.285	Large change^*∗∗*^	−0.61	0.021^‡^	−1	−0.175	Large change^*∗∗*^
CAS	−0.68	0.01^‡^	−1	−0.245	Large change^*∗∗*^	−0.57	0.031^‡^	−1	−0.135	Moderate change^*∗*^
PRPS	0.54	0.041^‡^	0.105	0.975	Moderate change^*∗*^	0.43	0.104	0.005	0.865	—

CBS: catherine bergego scale, CAS: clinical assessment for spontaneity, PRPS: pittsburgh rehabilitation participation scale. ^†^*p* < 0.01, ^‡^*p* < 0.05 were considered statistically significant. Small change; tau ＜0.20, ^*∗*^moderate change; 0.20 ≦ tau ＜ 0.60, ^*∗∗*^large change; 0.60 ≦ tau ＜0.80, ^*∗∗∗*^very large change; tau ≧0.80.

**Table 4:** Correlation analysis between the outcomes.

	CBS				CAS			
	r	*p* value	95% CI		r	*p* value	95% CI	
CBS	—	—	—	—	—	—	—	—
CAS	0.96	^*∗∗*^ *p* < 0.001	0.909	0.979	—	—	—	—
PRPS	−0.93	^*∗∗*^ *p* < 0.001	−0.968	−0.863	−0.96	^*∗∗*^ *p* < 0.001	−0.981	−0.917

CBS: catherine bergego scale, CAS: clinical assessment for spontaneity, PRPS: pittsburgh rehabilitation participation scale. ^*∗∗*^*p* < 0.01 and ^*∗*^*p* < 0.05 were considered statistically significant.
